# Novel Integrated Technology of Pixelized Inorganic Scintillator Wafers for X-Rays and Neutron Detection

**DOI:** 10.3390/molecules31122013

**Published:** 2026-06-09

**Authors:** Petr S. Sokolov, Lydia V. Ermakova, Aliaksei G. Bondarau, Petr V. Karpyuk, Valentina G. Smyslova, Alexey M. Sergeev, Ilia Y. Komendo, Vitaly A. Mechinsky, Elizaveta A. Borisevich, Andrey V. Popov, Dmitriy V. Sosnov, Mikhail V. Korzhik

**Affiliations:** 1 National Research Centre «Kurchatov Institute», 123098 Moscow, Russia; ermakova_lv@nrcki.ru (L.V.E.); karpyuk_pv@nrcki.ru (P.V.K.); smyslova_vg@nrcki.ru (V.G.S.); sergeev_am@nrcki.ru (A.M.S.); komendo_iyu@nrcki.ru (I.Y.K.); vitaly.mechinsky@gmail.com (V.A.M.); 2 Institute for Nuclear Problems, Belarus State University, 220030 Minsk, Belarus; a.bondarev.by@gmail.com (A.G.B.); gapovaknopka@mail.ru (E.A.B.); 3 Department of Chemistry and Technology of Crystals, Mendeleev University of Chemical Technology of Russia, 125047 Moscow, Russia; 4 Fabrika RTT LLC, 107078 Moscow, Russia; popov@frtt.ru (A.V.P.); sosnov@frtt.ru (D.V.S.)

**Keywords:** additive manufacturing, ceramics, garnet, scintillator, stereolithography

## Abstract

Pixelated detectors based on inorganic scintillation materials are widely used in radiation detection systems for medical imaging and many other fields of science and technology. A substantial application is X-ray scanning using flat-panel detectors (FPDs) for both fluorography and mammography. In this article, the detection properties of the monolithic planar ceramic scintillation elements are reported for the first time. A high-light yield (Gd,Y)_3_Al_2_Ga_3_O_12_:Ce,Mg garnet-type scintillation material was used to form square-shaped pixels, while a material of similar composition was used as a substrate. Green bodies were successfully fabricated by a digital light processing (DLP) 3D printing method. Subsequent debinding and pressureless high-temperature sintering resulted in composite elements consisting of two layers with different chemical compositions. The lower bulk layer consisted of transparent, non-luminescent garnet, whereas the upper pixelated layer, with pixel dimensions of 230 × 230 µm, was made of scintillation material. The spatial resolution of the matrices under UV light and alpha-particle excitation was evaluated. It was confirmed that the spatial resolution of the matrices produced by the developed technology is approximately 0.4 times the pixel size. The proven ability of the integrated technology of inorganic scintillation matrix production opens the way for future improvement in spatial resolution through optimizing the printed pixel dimensions.

## 1. Introduction

Nowadays, medical imaging technologies based on flat-panel X-ray detectors (FPDs) are widely used [1,2,3]. Two major FPD technical solutions have been implemented: direct conversion of ionizing radiation deposited energy into charge and indirect converters of X-rays or other particles into light by a scintillator and then into proportional charge by a photosensor. Afterwards, analog signals are acquired and digitized by a computer and can be used for analysis.

A breakthrough innovation in scintillation elements for FPDs was the use of thin detector plates made of columnar CsI(Tl) [4,5,6], as well as plates based on the Gd_2_O_2_S:Tb (GOS) composite [5,6,7]. The drawback of the columnar detector element is the low effective density of the material, which is equal to 0.7 times the density of a single crystal [8]. Moreover, the structure of the columnar element has a highly developed surface which, given the relatively high hygroscopicity of the material, imposes strict requirements for the packaging of such elements in matrices. GOS scintillation ceramics have a high scintillation yield and are used in powder form to create scintillation composites [9]. However, such ceramics are opaque and so are the powder particles made from them; therefore, the scintillation yield from a composite of a binder and GOS powder drops quickly with increasing plate thickness. Currently, promising composite detectors for X-ray imaging are based on glass and ZnS:Ag scintillator powder [10] or GAGG:Ce scintillator powder with a polysiloxane polymer binder [11]. Another area of application of FPDs, in the presence of Li, B, or Gd ions in the scintillator material, is the detection of thermal neutrons [12,13,14,15]. Moreover, research and development of multimodal detector elements for FPD have also progressed. A novel composite scintillator made of Li_6_Gd(BO_3_)_3_:Ce powder and polymethyl methacrylate for X-ray and thermal neutron detection was recently announced [16]. It exhibits blue light at 416 nm under UV excitation, shows performance superior to that of commercial CsI:Na under X-ray excitation, and provides a light yield of 21,000 photons/thermal neutron under thermal neutron irradiation. A common disadvantage of composite materials is the translucency of the detector layer. This precludes the production of relatively thick detector layers. This problem can be resolved by using the scintillation thin films deposited on the substrates of the same material [17,18]. However, thin films have a low stopping power for ionizing radiation, which results in longer acquisition times during detection.

A series of compounds with a garnet structure having compositions of (Gd,Y,Ce,Tb)_3_(Al,Ga)_5_O_12_ (GYAGG:Ce,Tb) can be obtained in transparent form as both single crystals and ceramics [19,20]. They are characterized by high scintillation yield when detecting X-ray and gamma-radiation [21,22]. GYAGG:Ce contains ^155^Gd and ^157^Gd isotopes, which have a thermal neutron capture cross-section size of 61,000 b and 255,000 b, respectively [23]. The material composition includes Gd^3+^ ions, of which its natural mixture has the highest neutron absorption cross-section [24]. Powders from grounded ceramics allow for the creation of neutron-sensitive screens with high performance characteristics [25].

A transition from traditional methods of composite and ceramic fabrication to digital production allows not only for the optimization of geometry but also for a programmable design of the internal architecture of composite and ceramic bodies, which is crucial for the development of next-generation scintillation detectors and laser media.

In the field of inorganic scintillation and luminescence materials with garnet structures, the most impressive results are obtained by various 3D printing methods for transparent YAG [26,27], LuAG:Ce [28,29], GYAGG:Ce [30], and GAGG:Ce [31] fine and bulk ceramics. For tasks requiring submicron precision, the two-photon polymerization (TPP) method is used, which allows for the fabrication of microscopic optical components with a resolution of less than 1 μm. The use of nanoscale particles and special precursors makes it possible to 3D print transparent ceramic structures of YAG:Nd [32], which retain high transparency after heat treatment even at extremely small sizes.

In this study, we focused on developing the fundamentals for the fabrication of monolithic detector elements consisting of scintillation pixels and a substrate obtained using a ceramic method. For the first time, monolithic ceramic pixelated detector elements were produced on a substrate made of the same material, with the breakthrough being the use of 3D stereolithographic printing to produce green bodies. High spatial resolution was demonstrated in both ultraviolet and alpha-particle measurements.

## 2. Results and Discussion

**Figure 1:**
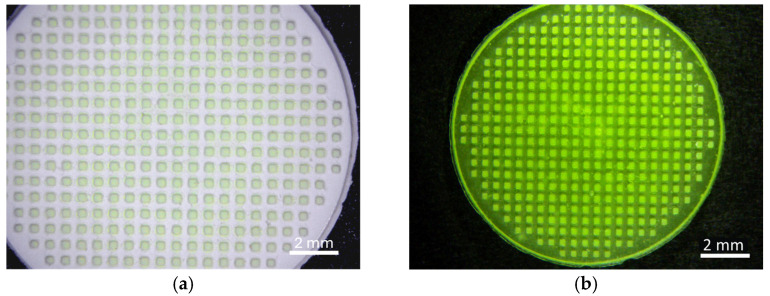
Optical photographs of the pixelated structure: (**a**) GYAGG:Ce and GYAGG/HDDA composite after DLP 3D printing; (**b**) ceramics after burnout and high-temperature sintering on a black paper substrate.

The representative views of green bodies and ceramic matrices obtained by optic microscopy are presented in Figure 1a and Figure 1b, respectively. The images show an almost defect-free pixelated structure both at the green body stage after DLP 3D printing and after debinding and high-temperature sintering, while the geometry of the array is well preserved. The average pixel tip size in the green body is 320 ± 10 μm with gaps of 320 ± 10 μm, which corresponds well with the initial model; the pixel bases are slightly wider due to the DLP 3D printing peculiarities. Due to shrinkage during high-temperature sintering in ceramics, the pixel size decreases to 230 ± 10 μm with gaps of 250 ± 15 μm. It is also seen that the ceramic base remains transparent and light yellow, which is probably due to the diffusion of cerium from pixels into the substrate volume. The measured relative density of ceramics is about 100%.

The ceramic microstructure was studied. GYAGG:Ce demonstrates an average grain size of 5.5 ± 1.0 μm, while GYAGG exhibits an increased grain size of 15 ± 1 μm due to 100 ppm of Mg acting as a sintering aid. The porosity of ceramics is less than 0.4%.

**Figure 2:**
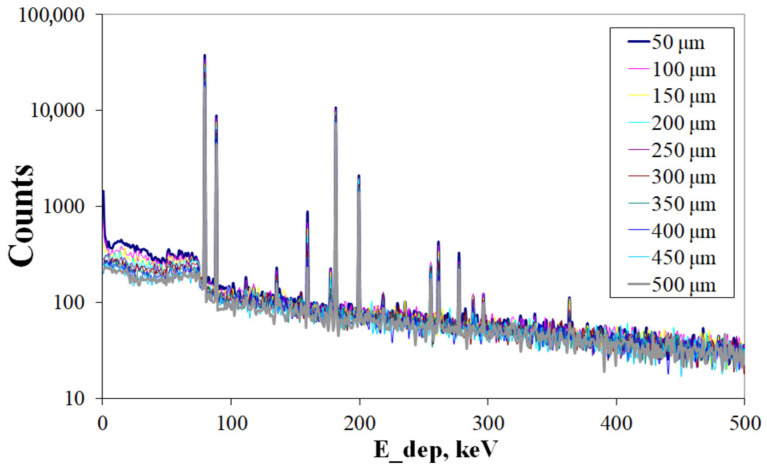
The spectra of the energy deposition in the sample in the range of up to 500 keV in GYAGG. The pixel cross-section varied from 50 × 50 μm^2^ to 500 × 500 μm^2^.

Figure 2 shows the results of the simulation by the Geant4 [33] package. The modeling aimed to evaluate cross-talk between the substrate and pixels. In the model, the beam of thermal neutrons (E_n_ = 0.0253 eV) with a diameter of 2 μm bombarded the center of the pixel on the substrate plate from the top. The pixel cross-section varied from 50 × 50 μm^2^ to 500 × 500 μm^2^, and the spectra of the energy deposition in the sample were determined by registering secondary particles leaving the pixel. The number of neutrons shooting out of the target was 10^6^.

**Figure 3:**
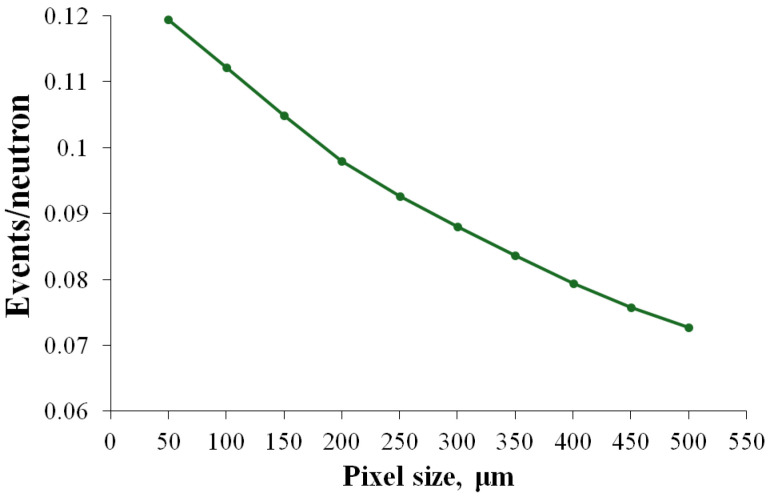
The number of events of secondary particles leaving the plate from the pixel provided that they have released energy in the plate above 20 keV.

Based on the obtained spectra, the number of events of secondary particles leaving the plate from the pixel was calculated provided that they released energy in the GYAGG disk above 20 keV. The obtained results, normalized by one primary neutron, are shown in Figure 3.

The mean free path of conversion electrons in this material is about 30 μm, which is fairly close to the photo-electrons created by the same energy. This means that even if the interaction occurs near the edge of the pixel, there is no cross-talk effect due to the photon migration between pixels. Moreover, the cross-talk can be easily suppressed by using, for example, a BaSO_4_ reflector in the space in between pixels. Therefore, a minimal cross-section of the acceptable pixel for both X-ray and thermal neutron detection is about 30 μm by 30 μm. This is sufficient for many applications in X-ray and neutron radiography. Moreover, pixels can be thicker to provide high stopping power for radiation, but spatial resolution is determined by the pixel cross-section.

**Figure 4:**
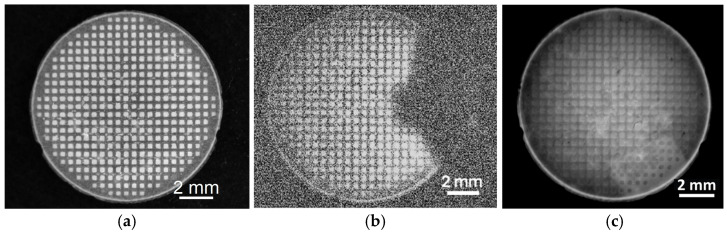
Images of the matrix excited by (**a**) a UV LED, (**b**) alpha-particles from a ^241^Am source, and thermal neutrons (**c**).

Figure 4a shows the image of the matrix under UV LED excitation. Figure 4b depicts the sum of 30 images of the matrix under alpha-particle excitation with an exposure of 600 s each obtained using IRIS software Version 5.59 [34]. The intensity variation in the latter image is due to a decrease in the alpha-particle flux according to the 1/r^2^ law, where r is the distance from the source to the pixel. For the excitation by ionizing radiation, alpha-particles with an energy of 5.4 MeV were selected. They provide intense ionization within a depth of several micrometers in both the pixels and the substrate. Also, the excitation by alpha-particles mimics excitation by low-energy X-rays; the length of their ionization tracks are comparable to the tracks of photo-electrons created by the X-rays. As for neutrons, the scintillator response due to the coinciding-in-time transitions to the ground state from the lowest excited energy levels of the ^156,158^Gd nuclei (X-rays~44 keV) and internal conversion electrons (~33 keV) are of particular interest [35]. In addition, the matrix was irradiated with monochromatic neutrons with an energy of 0.0818 eV with a flux of 4 × 10^6^ n/cm^2^. Exposure time was chosen to be 300 s in the latter case.

**Figure 5:**
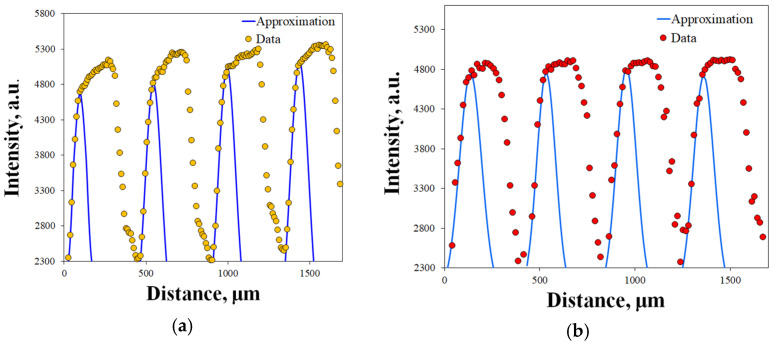
Average photometric profiles of the pixel matrix and the corresponding Gaussian fits under (**a**) UV radiation and (**b**) alpha-particle excitation.

By the means of ImageJ software Version 1.51j8 [36], based on the pictures obtained under UV (Figure 4a) and alpha-particles (Figure 4b), average photometric pixel profiles have been obtained (Figure 5). At the same time, the average pixel size was calculated, and it is equal to 229 ± 6 μm. Next, the increasing part of the averaged profile of each pixel was approximated by the Gaussian function and the standard deviation was doubled, which determined the spatial resolution of the pixel structure; the calculation method is considered in [37] and the determination of the spatial resolution is discussed in [38].

Furthermore, the ratio of the spatial resolution of a pixel to its size (230 μm) was calculated, and the values obtained were averaged over the number of pixels involved in the calculations. The final value of the ratio of the pixel’s spatial resolution to its size turned out to be about 0.41 when the matrix was excited by UV radiation and about 0.44 when the matrix was excited by alpha-particles from the ^241^Am source. A close intensity modulation depth in both cases suggests that the substrate plate does not scintillate. Therefore, such an element can provide reasonable-quality images even without filling the space in between pixels. During neutron measurements, the matrix structure was easily recognized. The matrix was fixed using an adhesive in several points to an Al plate. These parts appear as lighter areas in the bottom right part of Figure 4c.

## 3. Materials and Methods

### 3.1. Initial Materials and Powder Synthesis

Metallic gallium (Ga), boehmite (AlO(OH)), and high-purity oxides of yttrium (Y_2_O_3_) and gadolinium (Gd_2_O_3_), as well as cerium nitrate (Ce(NO_3_)_3_), were used as starting materials to synthesize the initial fine multicomponent oxide powders with a cubic garnet-type crystal structure (*Ia-3d*, #230). GYAGG:Ce and GYAGG powders were synthesized by co-precipitation from nitrate solutions of the corresponding elements using ammonium bicarbonate (NH_4_HCO_3_) as the precipitating agent. The dried precipitates were calcined at the following temperatures: 1300 °C for GYAGG:Ce and 1250 °C for GYAGG for 2 h. The powders were then milled down in a planetary ball mill.

Magnesium oxide (MgO) was added to the GYAGG powder as a highly diluted Mg(NO_3_)_2_ solution to obtain a Mg concentration of 100–200 ppm in the powder, and the mixture was mechanically mixed using grinding media. Prior to suspension preparation, the powders were dried at 70 °C in a laboratory oven to remove residual moisture.

**Figure 6:**
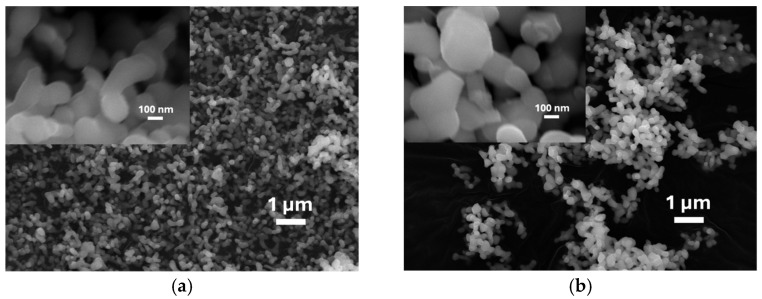
SEM images of initial powders: (**a**) GYAGG; (**b**) GYAGG:Ce.

**Figure 7:**
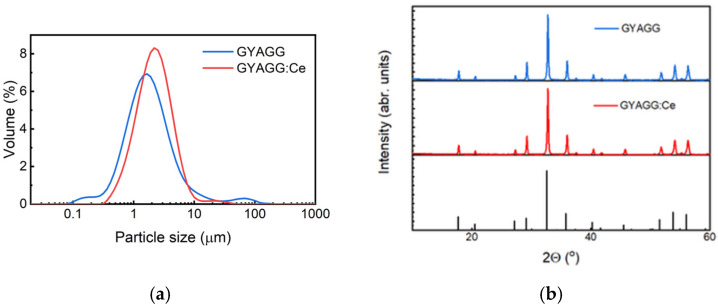
Initial powder characteristics: (**a**) particle size distributions; (**b**) X-ray diffraction patterns, the blue and red line is experimental pattern for undoped GYAGG and Ce-activated GYAGG powders, respectively. Below black lines are Bragg peaks positions for garnet crystal structure.

The specific surface area and porosity of the multicomponent powders of complex oxides with a garnet structure were measured using a TriStar 3000 analyzer (Micromeritics, Norcross, GA, USA) by BET and BJH methods, respectively. The measured specific surface areas of GYAGG:Ce and GYAGG powders were 4.5 and 5.8 m^2^/g, respectively. The porosity values of such powders were 0.007 and 0.008 cm^3^/g. The morphology of the primary powder particles (Figure 6) was analyzed using a JSM 7100f scanning electron microscope (Jeol, Akishima, Tokyo). GYAGG consists of agglomerated, predominantly rounded nanoparticles with a size of 30–70 nm (Figure 6a). GYAGG:Ce, calcined at the higher temperature, exhibits larger particles of 50–120 nm with a more distinct faceted morphology (Figure 6b). The particle size distribution (Figure 7a) was measured by a laser diffraction on a MasterSizer 2000 (Malvern Instruments, Malvern, UK). The phase purity of the initial garnet powders was confirmed by X-ray diffraction on a D2 Phaser (Bruker, Billerica, MA, USA) using Cu K_α1,2_ radiation (Figure 7b). Thus, both initial garnet-type powders have a comparable specific surface area, micromorphology, and average aggregate size (d_50_), which makes it possible to achieve similar sintering capacities (ceramic shrinkage during high-temperature sintering).

### 3.2. Slurry Preparation and 3D Printing

The commercially available 1,6-hexanediol diacrylate (HDDA, technical grade, Sigma-Aldrich, Saint Louis, MO, USA), known for its controlled polymerization kinetics and low viscosity (6–8 mPa·s at 25 °C), was used as the monomer [39]. The photoinitiator diphenyl (2,4,6-trimethylbenzoyl) phosphine oxide was used to initiate the radical polymerization reaction. The ceramic suspension was stabilized with a previously experimentally selected phosphorus-free DISPERBYK dispersant [40].

The preparation of photocurable suspensions began with mixing the acrylate monomer, photoinitiator, and a dispersant. Then the garnet oxide powders were added step-by-step until the solid content reached 40 vol.% (about 80 wt.%). A homogeneous suspension was prepared using a vacuum mixer and ZrO_2_ grinding beads in transparent polypropylene vials.

The rheology of the photocurable suspensions was studied using a Physica MCR 52 rheometer (Anton Paar, Graz, Austria) in a parallel plate geometry with a measuring disk diameter of 25 mm and a gap of 0.2 mm. The viscosity was measured as a function of the shear rate in the range of 10–200 s^−1^ at a fixed temperature of 20.0 °C. The dependence of the shear stress on the shear rate was approximated by the Herschel–Bulkley equation [41]:

τ = τ_0_ + Kγ^n^, (1)

where τ_0_ is the yield strength, K is the consistency index, and n is the flow behavior index.

The depth of photopolymerization was assessed using the DLP 3D printer Ember (Autodesk, San Francisco, CA, USA, λ = 405 nm, E = 11 mJ/cm^2^) curing single-layer disks at different exposure doses, the thickness of which was determined on a micrometer. Based on these data, plots showing the dependence of the cured layer thickness on the energy dose were constructed to determine the optimal printing parameters. The measured values were approximated using the Jacobs model derived from the Lambert–Bouguer–Baer law [42]:

C_d_ = D_p_ ln(E_max_/E_c_) (2)

where C_d_ is the cure depth; D_p_ is the penetration depth; E_max_ is the maximum exposure energy; and E_c_ is the critical (lowest) exposure energy required to activate monomer polymerization. The measured viscosity and cure depth of the photosensitive suspensions are given in the Supplementary Materials. All measurements were carried out at room temperature.

A commercially available, low-cost, and simple desktop DLP 3D printer, Photon Ultra (Anycubic, Shenzhen, China, λ = 405 nm, E = 2.35 mJ/cm^2^), was used to form dual-layer structured green bodies. Such a printer implements the method using a bottom-up vat polymerization configuration. The light intensity of the UV projector was measured with a UV light meter Model 222 (G&R Labs, Santa Clara, CA, USA). The nominal resolution of the printer along the X and Y axes was 80 × 80 μm.

**Figure 8:**
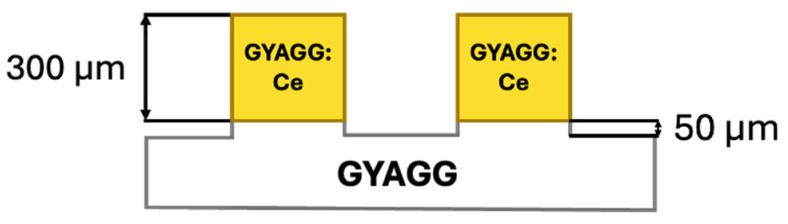
Schematic representation of the layer configuration of the CAD model.

The 3D model was built using the KOMPAS-3D computer-aided design software Version 23 (ASCON, Saint Petersburg, Russia). See the stl-file of the pixelate matrix in the Supplementary Materials. It consisted of a bulk colorless substrate with a diameter of 15 mm and a thickness of 1 mm, on which rectangular pixels were located with a certain gap between them. The CAD model had 320 × 320 × 350 μm pixels with a gap of 320 μm between them. The file was sliced using Photon Workshop software Version 4.1.1 (Anycubic, China). The bottom layer of the pixels and the substrate consisted of non-activated, non-luminescent (“blanc”) compositions with GYAGG:Mg powder, while the yellow pixels consisted of the ceria-activated garnet powder GYAGG:Ce (the scintillation material). To reduce the diffusion of cerium ions from the pixels into the bulk substrate during ceramic sintering at high temperature, the pixels had a complex structure (Figure 8). The bottom 50 μm layer served as part of a reflective substrate, while the remaining volume consisted of cubic scintillation pixels. Three-dimensional printing was performed sequentially by replacing the resin vats with suspensions of different compositions.

After printing, the green bodies were washed in HDDA in a laboratory ultrasonic bath, dried, and post-cured under UV light on both sides. The removal of the organic binder from the green bodies was carried out in an Ar/H_2_ atmosphere (95/5%) by heating from room temperature up to 500 °C at a rate of 0.6 °C/min. Then, the resulting brown bodies were fired into the air in a muffle furnace. Finally, bilayer bodies (GYAGG:Ce/GYAGG) were sintered into a dense ceramic matrix in an oxygen flow in a tubular furnace for 2 h at 1720 °C. White YAG ceramics were used as a sintering substrate.

### 3.3. Monolithic Scintillation Element Characterization

**Figure 9:**
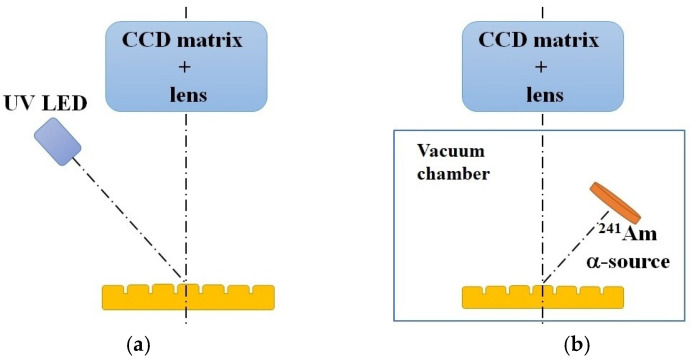
The schematic representation of the setup used to measure the spatial resolution of the matrix under (**a**) UV and (**b**) alpha-particle excitation.

To measure the spatial resolution of the pixelated matrix, the self-made purpose-designed setups were used (Figure 9a,b) at room temperature. In the first case, the setup consisted of a CCD matrix, a lens with a green filter, and a UV LED (265–285 nm) [43] placed in a light-isolated box. In the second case, alpha-particles from a ^241^Am source with activity 10^4^ Bq in 4π, which was positioned at a distance of 1 cm and at approximately a 45-degree angle relative to the normal to the surface of the matrix, were used. The alpha-particle source and the matrix were placed in a vacuum chamber (Figure 9b) to avoid energy losses of the alpha-particles in the air.

**Figure 10:**
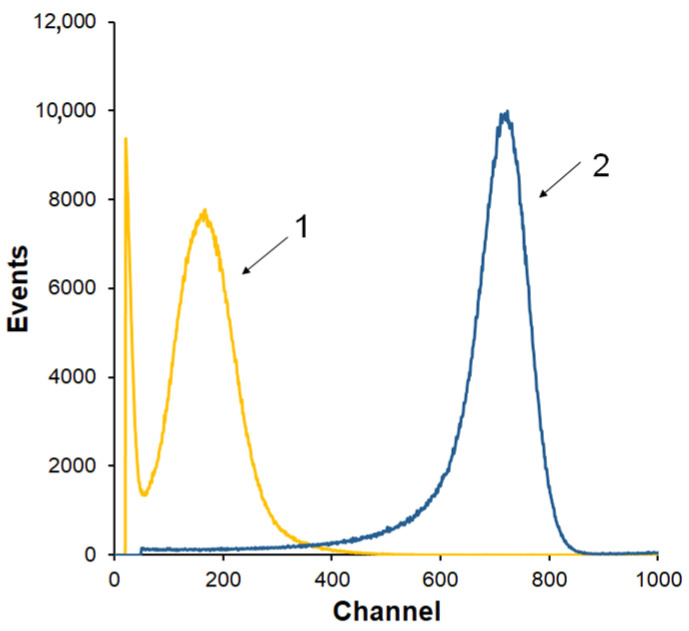
Pulse height spectra of alpha-particles (5.4 MeV) measured with matrix (1) and reference YAG:Ce (2).

GYAGG:Ce,Mg scintillation ceramics exhibit a light yield of approximately 40,000 ph/MeV [30]. Figure 10 demonstrates a comparison of the pulse height spectra measured for a reference sample of Y_3_Al_5_O_12_:Ce (YAG:Ce, 24,000 ph/MeV) and a matrix under alpha-particle excitation. Both measurements were performed without immersion liquid in between the sample and the photoreceiver window of the XP2020 photomultiplier (Philips, Cambridge, MA, USA). During the measurements, pixels were at the top, so additional light losses were created by the substrate between the pixels and PMT window. 

## 4. Conclusions

A novel, cost-effective, and relatively straightforward approach to producing a two-layer inorganic scintillator ceramic material, consisting of a transparent GYAGG:Mg substrate with GYAGG:Ce,Mg pixels, is presented. This matrix can be utilized in planar detectors for various ionizing radiations. This is the first experimental validation of the idea that a structured pixelated detector with high spatial resolution can be created using inexpensive and readily accessible equipment. A pixel spatial resolution-to-pixel size ratio of approximately 0.4 suggests that lasers or more sophisticated DLP 3D printers, capable of printing with a high lateral resolution of 30–50 microns, can demonstrate spatial resolutions of around 10–20 microns.

## Data Availability

Any primary data and other results supporting the conclusions of this study are available from the corresponding author upon reasonable request.

## Supplementary Materials

The following supporting information can be downloaded at: https://www.mdpi.com/article/10.3390/molecules31122013/s1, Figure S1: Viscosity versus shear rate for the photocurable HDDA-based suspensions with GYAGG:Ce and GYAGG fine powders (solid loading 35 vol.%) at 20.0 °C; Figure S2: Cure depth versus energy dose for the photocurable HDDA-based suspensions with GYAGG:Ce and GYAGG fine powders (solid loading 35 vol.%) at room conditions. Stl-file with computer model of pixeled matrix.

## Abbreviations

The following abbreviations are used in this manuscript:

BET	Brunauer–Emmett–Teller
BJH	Barrett–Joyner–Halenda
CCD	Charge-coupled device
DLP	Digital light processing
FPDs	Flat-panel detectors
GAGG	Gadolinium aluminum gallium garnet
GYAGG:Ce	Cerium-doped gadolinium–yttrium aluminum gallium garnet
HDDA	1,6-hexanediol diacrylate
LED	Light-emitting diode
LuAG	Lutecium aluminum garnet
UV	Ultraviolet
YAG	Yttrium aluminum garnet
