# Supplementary material for: Novel Integrated Technology of Pixelized Inorganic Scintillator Wafers for X-Rays and Neutron Detection
Source: Molecules. 2026 Jun 9;31(12):2013. doi: 10.3390/molecules31122013 (PMC13305111; doi:10.3390/molecules31122013)
Supplement: Supplementary file 1 [file molecules-31-02013-s001.zip › Supplementary materials.pdf]

Supplementary materials for

Novel Integrated Technology of Pixelized Inorganic Scintillator Wafers for X-Rays and Neutron Detection

Petr S. Sokolov [1,*], Lydia V. Ermakova [1], Aliaksei G. Bondarau [2], Petr V. Karpyuk [1], Valentina G. Smyslova [1], Alexey M. Sergeev [1,3], Ilia Y. Komendo [1,3], Vitaly A. Mechinsky [1,2], Elizaveta A. Borisevich [2], Andrey V. Popov [4], Dmitriy V. Sosnov [4] and Mikhail V. Korzhik [1,2,*]

1 National Research Centre «Kurchatov Institute», 123098 Moscow, Russia; ermakova_lv@nrcki.ru (L.V.E.);
karpyuk_pv@nrcki.ru (P.V.K.); smyslova_vg@nrcki.ru (V.G.S.); sergeev_am@nrcki.ru (A.M.S.); komendo_iyu@nrcki.ru (I.Y.K.); vitaly.mechinsky@gmail.com (V.A.M.)
2 Institute for Nuclear Problems, Belarus State University, 220030 Minsk, Belarus; a.bondarev.by@gmail.com (A.G.B.); gapovaknopka@mail.ru (E.A.B.)
3 Department of Chemistry and Technology of Crystals, Mendeleev University of Chemical Technology of Russia, 125047 Moscow, Russia
4 Fabrika RTT LLC, 107078 Moscow, Russia; popov@frtt.ru (A.V.P.); sosnov@frtt.ru (D.V.S.)
* Correspondence: sokolov-petr@yandex.ru or sokolov_ps@nrcki.ru (P.S.S.); korjikmikhail@gmail.com (M.V.K.)

This PDF file includes:

Figures S1, S2

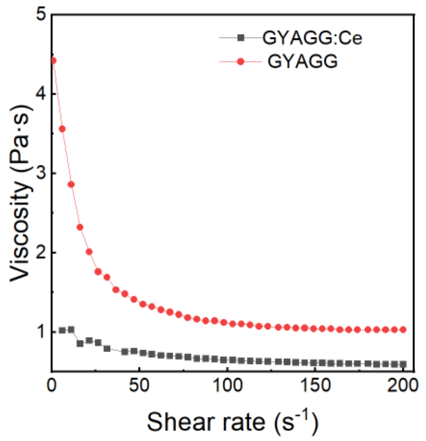

Figure S1. Viscosity *versus* shear rate for the photocurable HDDA-based suspensions with GYAGG:Ce and GYAGG fine powders (solid loading 35 vol.%) at 20.0 °C.

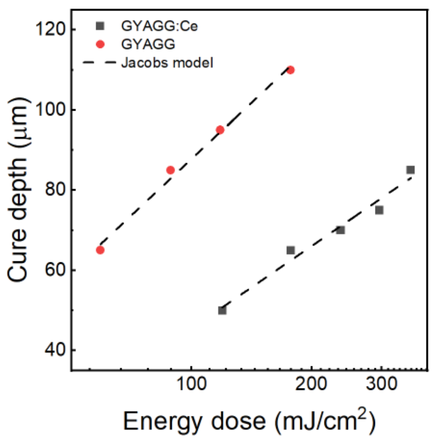

Figure S2. Cure depth *versus* energy dose for the photocurable HDDA-based suspensions with GYAGG:Ce and GYAGG fine powders (solid loading 35 vol.%) at room conditions.
